# 5,6-Dihydro-1,10-phenanthroline-1,10-diium μ-oxido-bis­[penta­fluoridotantalate(V)]

**DOI:** 10.1107/S1600536812014742

**Published:** 2012-04-13

**Authors:** Zhao-Hui Meng, Yu-Quan Feng, Xin-Feng Chen

**Affiliations:** a College of Chemistry and Pharmacy Engineering, Nanyang Normal University, Nanyang 473061, People’s Republic of China

## Abstract

In the title compound, (C_12_H_12_N_2_)[Ta_2_F_10_O], the doubly protonated 5,6-dihydro-1,10-phenantroline-1,10-diium cation is located on a twofold rotation axis, whereas the isolated [Ta_2_OF_10_]^2−^ dianion has -1 symmetry. In the so far unknown dianion, the symmetry-related Ta^V^ atoms are octa­hedrally coordinated by five F atoms and a bridging O atom, the latter being located on an inversion centre. The two pyridine rings in the cation make a dihedral angle of 22.8 (4)°. The cations and dianions are arranged in layers parallel to (100) and are connected through N—H⋯F and C—H⋯F hydrogen-bonding inter­actions into a three-dimensional structure.

## Related literature

For structure–property relations of metal oxyfluorides, see: Hagerman & Poeppelmeier (1995 ▶); Halasyamani & Poeppelmeier (1998 ▶); Welk *et al.* (2002 ▶).
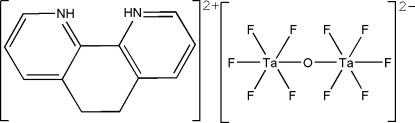


## Experimental

### 

#### Crystal data

(C_12_H_12_N_2_)[Ta_2_F_10_O]
*M* *_r_* = 752.14
Monoclinic, 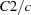

*a* = 13.536 (2) Å
*b* = 11.3031 (17) Å
*c* = 11.5316 (17) Å
β = 90.093 (2)°
*V* = 1764.4 (5) Å^3^
*Z* = 4
Mo *K*α radiation
μ = 12.50 mm^−1^
*T* = 296 K
0.21 × 0.20 × 0.17 mm

#### Data collection

Bruker APEXII CCD diffractometer
Absorption correction: multi-scan (*SADABS*; Bruker, 2008 ▶) *T*_min_ = 0.179, *T*_max_ = 0.225
4738 measured reflections
1725 independent reflections
1573 reflections with *I* > 2σ(*I*)
*R*_int_ = 0.029

#### Refinement

*R*[*F* ^2^ > 2σ(*F* ^2^)] = 0.028
*wR*(*F* ^2^) = 0.072
*S* = 1.05
1725 reflections
124 parameters
H-atom parameters constrained
Δρ_max_ = 1.96 e Å^−3^
Δρ_min_ = −1.14 e Å^−3^

### 

Data collection: *APEX2* (Bruker, 2008 ▶); cell refinement: *SAINT* (Bruker, 2008 ▶); data reduction: *SAINT*; program(s) used to solve structure: *SHELXS97* (Sheldrick, 2008 ▶); program(s) used to refine structure: *SHELXL97* (Sheldrick, 2008 ▶); molecular graphics: *SHELXTL* (Sheldrick, 2008 ▶); software used to prepare material for publication: *SHELXTL*.

## Supplementary Material

Crystal structure: contains datablock(s) I, global. DOI: 10.1107/S1600536812014742/wm2602sup1.cif

Structure factors: contains datablock(s) I. DOI: 10.1107/S1600536812014742/wm2602Isup2.hkl

Additional supplementary materials:  crystallographic information; 3D view; checkCIF report

## Figures and Tables

**Table 1:** Selected bond lengths (Å)

Ta1—F4	1.877 (5)
Ta1—F5	1.886 (5)
Ta1—F1	1.886 (5)
Ta1—O1	1.8924 (3)
Ta1—F3	1.895 (4)
Ta1—F2	1.905 (4)

**Table 2:** Hydrogen-bond geometry (Å, °)

*D*—H⋯*A*	*D*—H	H⋯*A*	*D*⋯*A*	*D*—H⋯*A*
N1—H1*A*⋯F4^i^	0.86	2.45	3.114 (10)	135
C4—H4*A*⋯F1^ii^	0.93	2.26	3.066 (9)	145
C6—H6*A*⋯F3^iii^	0.97	2.28	3.219 (8)	163
C6—H6*B*⋯F5^iv^	0.97	2.45	3.268 (9)	142

Symmetry codes: (i) 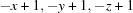; (ii) 
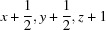
; (iii) 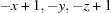; (iv) 
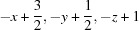
.

**Fig. 1.**
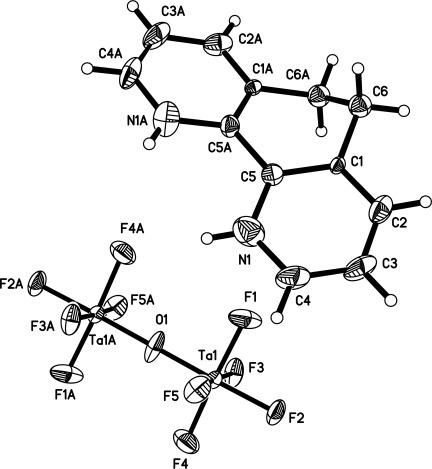
View of the title molecule with displacement ellipsoids drawn at the 30% probability level [symmetry code A: -x+2, y, -z+3/2].

**Fig. 2.**
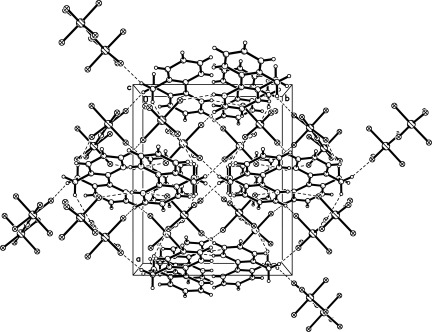
Crystal packing viewed along the c axis. Hydrogen bonding interactions are shown as dashed lines.

**Fig. 3.**
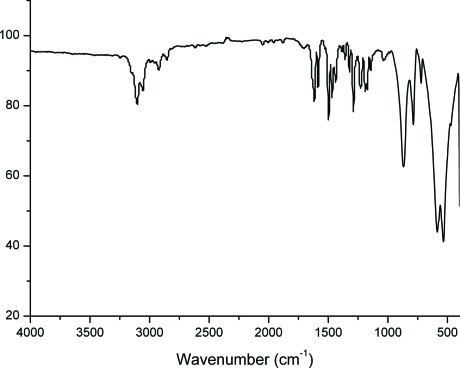
IR spectrum of the title compound.

## supplementary crystallographic information

### Comment

Metal oxyfluorides have received considerable attention in recent years due to their structure-related properties such as ferroelectricity, piezoelectricity and second-order nonlinear optical activity (Hagerman & Poeppelmeier, 1995; Halasyamani & Poeppelmeier, 1998; Welk *et al.*, 2002). In this article, we report on a new oxidofluoridotantalate with composition [C_12_H_12_N_2_][Ta_2_OF_10_] that was obtained by means of a two-step hydrothermal method.

The title compound (Fig. 1) contains one diprotonated 5,6-dihydro-1,10-phenantroline-1,10-diium cation (symmetry 2) and one [Ta_2_OF_10_]^2-^ dianion (symmetry 1). In the latter, the Ta^V^ ion is coordinated by five fluorine atoms and one oxygen atom, forming an octahedral coordination geometry. It is noteworthy that the title compound features the first oxidofluoridotantalate with composition [Ta_2_OF_10_]^2-^. The cation is not flat, as can be expected from the 5,6-dihydro bridging *sp*^3^ carbon atoms, with a dihedral angle of of 22.8 (4)° between the two pyridine rings. The cations and dianions are arranged in layers parallel to (100) and are connected through N—H···F and C—H···F hydrogen bonding interactions into a three-dimensional structure (Fig. 2).

It should be noted that the hydrothermal conditions make it possible that parts of the fluorine atoms are replaced by OH^-^ ions. To exclude the presence of the latter, additional characterisation methods were employed (see details in the experimental part). Moreover, IR spectroscopy revealed no inclusion of OH^-^ in the compound (Fig. 3).

### Experimental

All chemicals were of reagent grade quality obtained from commercial sources and were used without further purification. The title compound was obtained by using a two-step hydrothermal method in a 50 mL Teflon-lined autoclave. Firstly, 0.66 g Ta_2_O_5_ (1.5 mmol) was dissolved in 1.11 g HF (40*_wt_*%) (7.4 mmol) and heated to 453 K for 4 hours. After it was cooled, the solution was added into 0.90 mL H_3_PO_4_ (85*_wt_*%), 0.24 g 2,2'-bipyridine (1.5 mmol), 2.0 mL ethylene glycol and 1.0 mL H_2_O. Then the mixture was stirred for half an hour, and transferred into a Teflon-lined stainless steel autoclave (50 mL) and treated at 453 K for 7 days. After the mixture was slowly cooled to room temperature, yellow block-like crystals suitable for X-ray structure determination were obtained. It worth noting that the reaction of 2,2'-bipyridine and ethylene glycol produced the 5,6-dihydro-1,10-phenantroline ligand. The chemical composition of the title compound was confirmed by EDS and elemental analysis. The results of EDS indicate the presence of the elements Ta, F, O, C and N. The Ta composition was quantified by ICP-OES: *Anal.*/*Calcd* (%): Ta: 48.59/48.12. C, H, and N analysis was performed on a PerkinElmer 2400II elemental analyzer. *Anal.*/*Calcd* (%): C, 19.16; H, 1.61; N,3.72 %. Found: C, 19.63; H, 1.94; N, 3.17 %. IR (KBr, cm^-1^) (Fig. 3): 3110, 3057, 2920, 2861, 1621,1584,1494, 1457, 1431, 1367, 1330, 1282, 1234, 1181, 1149, 1033, 869, 784, 715, 593 and 535.

### Refinement

The H atoms bonded to C and N were positioned geometrically and refined using a riding model, with C—H = 0.93 Å for H atoms bound to *sp*^2^ C atoms, and 0.97 Å for H atoms bound to *sp*^3^ C atoms, and with N—H = 0.86 Å, and with *U*_iso_(H) = 1.2 (1.5) times *U*_eq_(C), and *U*_iso_(H) = 1.2 times *U*_eq_(N), respectively. The highest and lowest remaining electron density was located 0.84 Å and 0.72 Å from atom Ta1.

### Crystal data

**Table:**

(C_12_H_12_N_2_)[Ta_2_F_10_O]	*F*(000) = 1368
*M**_r_* = 752.14	*D*_x_ = 2.831 Mg m^−^^3^
Monoclinic, *C*2/*c*	Mo *K*α radiation, λ = 0.71073 Å
Hall symbol: -C 2yc	Cell parameters from 3156 reflections
*a* = 13.536 (2) Å	θ = 2.4–28.3°
*b* = 11.3031 (17) Å	µ = 12.50 mm^−^^1^
*c* = 11.5316 (17) Å	*T* = 296 K
β = 90.093 (2)°	Block, yellow
*V* = 1764.4 (5) Å^3^	0.21 × 0.20 × 0.17 mm
*Z* = 4	

### Data collection

**Table:**

Bruker APEXII CCD diffractometer	1725 independent reflections
Radiation source: fine-focus sealed tube	1573 reflections with *I* > 2σ(*I*)
Graphite monochromator	*R*_int_ = 0.029
φ and ω scans	θ_max_ = 26.0°, θ_min_ = 2.9°
Absorption correction: multi-scan (*SADABS*; Bruker, 2008)	*h* = −13→16
*T*_min_ = 0.179, *T*_max_ = 0.225	*k* = −13→13
4738 measured reflections	*l* = −14→12

### Refinement

**Table:**

Refinement on *F*^2^	Primary atom site location: structure-invariant direct methods
Least-squares matrix: full	Secondary atom site location: difference Fourier map
*R*[*F*^2^ > 2σ(*F*^2^)] = 0.028	Hydrogen site location: inferred from neighbouring sites
*wR*(*F*^2^) = 0.072	H-atom parameters constrained
*S* = 1.05	*w* = 1/[σ^2^(*F*_o_^2^) + (0.0278*P*)^2^ + 20.5568*P*] where *P* = (*F*_o_^2^ + 2*F*_c_^2^)/3
1725 reflections	(Δ/σ)_max_ < 0.001
124 parameters	Δρ_max_ = 1.96 e Å^−^^3^
0 restraints	Δρ_min_ = −1.14 e Å^−^^3^

### Special details

**Table:**

Geometry. All esds (except the esd in the dihedral angle between two l.s. planes) are estimated using the full covariance matrix. The cell esds are taken into account individually in the estimation of esds in distances, angles and torsion angles; correlations between esds in cell parameters are only used when they are defined by crystal symmetry. An approximate (isotropic) treatment of cell esds is used for estimating esds involving l.s. planes.

### Fractional atomic coordinates and isotropic or equivalent isotropic displacement parameters (Å^2^)

**Table:**

	*x*	*y*	*z*	*U*_iso_*/*U*_eq_	
Ta1	0.193168 (19)	0.18732 (2)	0.13666 (2)	0.03630 (12)	
C2	0.9169 (6)	0.2166 (8)	0.9660 (7)	0.0499 (19)	
H2A	0.9123	0.1448	1.0050	0.060*	
C1	0.9649 (4)	0.2220 (5)	0.8646 (5)	0.0210 (10)	
C5	0.9738 (5)	0.3254 (5)	0.8051 (5)	0.0332 (13)	
N1	0.9318 (6)	0.4270 (7)	0.8502 (7)	0.069 (2)	
H1A	0.9361	0.4931	0.8136	0.083*	
C4	0.8832 (7)	0.4207 (9)	0.9546 (7)	0.062 (2)	
H4A	0.8558	0.4890	0.9858	0.075*	
C3	0.8742 (7)	0.3164 (9)	1.0135 (7)	0.060 (2)	
H3A	0.8403	0.3127	1.0834	0.072*	
F1	0.2944 (5)	0.0732 (6)	0.1479 (5)	0.093 (2)	
F2	0.1355 (4)	0.1192 (5)	0.2716 (4)	0.0684 (14)	
F3	0.1159 (4)	0.0811 (4)	0.0475 (4)	0.0692 (15)	
F4	0.0908 (5)	0.2992 (5)	0.1343 (7)	0.0867 (19)	
F5	0.2665 (4)	0.2877 (5)	0.2351 (5)	0.0720 (15)	
O1	0.2500	0.2500	0.0000	0.082 (3)	
C6	1.0104 (5)	0.1139 (6)	0.8134 (6)	0.0408 (15)	
H6A	0.9827	0.0436	0.8490	0.049*	
H6B	1.0811	0.1141	0.8270	0.049*	

### Atomic displacement parameters (Å^2^)

**Table:**

	*U*^11^	*U*^22^	*U*^33^	*U*^12^	*U*^13^	*U*^23^
Ta1	0.03744 (18)	0.03940 (18)	0.03206 (18)	−0.00695 (11)	0.00427 (11)	−0.00056 (11)
C2	0.047 (4)	0.069 (5)	0.033 (4)	−0.008 (4)	0.001 (3)	0.012 (4)
C1	0.023 (3)	0.023 (2)	0.016 (2)	−0.001 (2)	0.004 (2)	0.002 (2)
C5	0.039 (3)	0.032 (3)	0.028 (3)	0.000 (3)	0.001 (3)	−0.004 (2)
N1	0.087 (5)	0.062 (5)	0.059 (4)	0.015 (4)	0.000 (4)	−0.007 (4)
C4	0.069 (6)	0.076 (6)	0.042 (4)	0.016 (5)	0.015 (4)	−0.022 (4)
C3	0.056 (5)	0.099 (7)	0.025 (4)	0.003 (4)	0.013 (3)	−0.012 (4)
F1	0.094 (4)	0.117 (5)	0.066 (3)	0.058 (4)	−0.006 (3)	−0.024 (3)
F2	0.097 (4)	0.066 (3)	0.042 (3)	−0.019 (3)	0.024 (3)	0.005 (2)
F3	0.095 (4)	0.065 (3)	0.048 (3)	−0.038 (3)	−0.010 (3)	0.002 (2)
F4	0.070 (4)	0.061 (3)	0.128 (6)	0.017 (3)	−0.009 (4)	0.007 (3)
F5	0.071 (3)	0.082 (4)	0.063 (3)	−0.031 (3)	0.001 (3)	−0.023 (3)
O1	0.106 (8)	0.095 (7)	0.045 (5)	−0.054 (6)	0.018 (5)	0.008 (5)
C6	0.043 (4)	0.031 (3)	0.048 (4)	0.002 (3)	0.005 (3)	0.004 (3)

### Geometric parameters (Å, º)

**Table:**

Ta1—F4	1.877 (5)	C5—N1	1.384 (9)
Ta1—F5	1.886 (5)	C5—C5^i^	1.455 (13)
Ta1—F1	1.886 (5)	N1—C4	1.374 (11)
Ta1—O1	1.8924 (3)	N1—H1A	0.8600
Ta1—F3	1.895 (4)	C4—C3	1.366 (13)
Ta1—F2	1.905 (4)	C4—H4A	0.9300
C2—C1	1.340 (9)	C3—H3A	0.9300
C2—C3	1.381 (12)	O1—Ta1^ii^	1.8924 (3)
C2—H2A	0.9300	C6—C6^i^	1.488 (14)
C1—C5	1.361 (8)	C6—H6A	0.9700
C1—C6	1.490 (8)	C6—H6B	0.9700
			
F4—Ta1—F5	89.5 (3)	C5—C1—C6	117.8 (5)
F4—Ta1—F1	176.8 (3)	C1—C5—N1	119.1 (6)
F5—Ta1—F1	89.3 (3)	C1—C5—C5^i^	119.0 (4)
F4—Ta1—O1	92.1 (2)	N1—C5—C5^i^	122.0 (5)
F5—Ta1—O1	93.55 (17)	C4—N1—C5	118.9 (8)
F1—Ta1—O1	91.0 (2)	C4—N1—H1A	120.5
F4—Ta1—F3	90.7 (3)	C5—N1—H1A	120.5
F5—Ta1—F3	175.8 (2)	C3—C4—N1	121.6 (8)
F1—Ta1—F3	90.2 (3)	C3—C4—H4A	119.2
O1—Ta1—F3	90.60 (15)	N1—C4—H4A	119.2
F4—Ta1—F2	88.9 (3)	C4—C3—C2	118.1 (7)
F5—Ta1—F2	88.1 (2)	C4—C3—H3A	121.0
F1—Ta1—F2	88.1 (3)	C2—C3—H3A	121.0
O1—Ta1—F2	178.07 (15)	Ta1^ii^—O1—Ta1	180.00 (2)
F3—Ta1—F2	87.7 (2)	C1—C6—C6^i^	108.2 (5)
C1—C2—C3	120.8 (7)	C1—C6—H6A	110.1
C1—C2—H2A	119.6	C6^i^—C6—H6A	110.1
C3—C2—H2A	119.6	C1—C6—H6B	110.1
C2—C1—C5	121.5 (6)	C6^i^—C6—H6B	110.1
C2—C1—C6	120.7 (6)	H6A—C6—H6B	108.4
			
C3—C2—C1—C5	0.4 (11)	C5^i^—C5—N1—C4	179.8 (8)
C3—C2—C1—C6	−179.6 (7)	C5—N1—C4—C3	−1.0 (13)
C2—C1—C5—N1	−0.4 (10)	N1—C4—C3—C2	1.0 (14)
C6—C1—C5—N1	179.6 (6)	C1—C2—C3—C4	−0.7 (13)
C2—C1—C5—C5^i^	−179.6 (8)	C2—C1—C6—C6^i^	138.0 (7)
C6—C1—C5—C5^i^	0.4 (10)	C5—C1—C6—C6^i^	−42.0 (9)
C1—C5—N1—C4	0.7 (11)		

Symmetry codes: (i) −*x*+2, *y*, −*z*+3/2; (ii) −*x*+1/2, −*y*+1/2, −*z*.

### Hydrogen-bond geometry (Å, º)

**Table:**

*D*—H···*A*	*D*—H	H···*A*	*D*···*A*	*D*—H···*A*
N1—H1*A*···F4^iii^	0.86	2.45	3.114 (10)	135
C4—H4*A*···F1^iv^	0.93	2.26	3.066 (9)	145
C6—H6*A*···F3^v^	0.97	2.28	3.219 (8)	163
C6—H6*B*···F5^vi^	0.97	2.45	3.268 (9)	142

Symmetry codes: (iii) −*x*+1, −*y*+1, −*z*+1; (iv) *x*+1/2, *y*+1/2, *z*+1; (v) −*x*+1, −*y*, −*z*+1; (vi) −*x*+3/2, −*y*+1/2, −*z*+1.
